# Reviving surgery with the smile, excitement, and Gemeinschaft concept: attempt at the Department of Surgery, Jikei University

**DOI:** 10.1515/iss-2019-0005

**Published:** 2019-06-22

**Authors:** Takao Ohki

**Affiliations:** Division of Vascular Surgery, Department of Surgery, The Jikei University School of Medicine, Tokyo, Japan

**Keywords:** excitement, lack of incentive, revival of surgery, surgeon shortage, surgical leadership

## Abstract

Between 1994 and 2004, the number of surgeons in Japan declined by 18%, whereas the total number of medical doctors increased by 30% during the same period. This was due to the fact that the younger generation avoided tough working environments with long working hours. We attempted to revive surgery by reintroducing the good old Japanese community as the model under the slogan of “intimate community with excitement and sense of secureness”. In the absence of financial incentives, we were able to recruit young staff, and the number of surgeons at Jikei University has increased by 28% over the last 12 years and currently we have 280 surgeons. Our experience showed that although the younger generation is conscious about quality of life and financial success, they also value excitement, friendship, and happiness, something we were able to provide without financial spending. However, our success may be an exception and cannot be generalized; therefore, we should continue to strive to improve the surgeon’s quality of life by creating a better working environment, including sustainable work hours and decent financial incentives.

## History of the Department of Surgery at Jikei University

The origin of the Department of Surgery at Jikei University can be traced back to the founder of Jikei University, Dr. Kanehiro Takagi. In 1880, Dr. Takagi received a diploma from the Royal College of Surgeons in the United Kingdom while studying at St Thomas’s Hospital in England, after which he returned to Japan as a 31-year-old surgeon and founded the Seiikai Medical Training Institute that would become the tailwind for Jikei University in 1881. At the beginning of the 20th century, surgery was performed at both Tokyo Jieikai Hospital and Tokyo Charity Hospital, and Professor Takagi who was also a Baron was the Medical Director and Surgical Supervisor at both institutes. Surgery at these hospitals flourished, and in 1973 the third Department of Surgery and then in 1985 Aoto surgery became independent, establishing the four surgical department system [1], [2].

In the 1990s, because the presence of multiple departments of surgery was difficult for patients to understand and because each department was competing and performing the same procedure at the same hospital and there were disadvantages in terms of personnel utilization, the need to merge the four independent departments and create a single large department system arose. With the resignation of Professor Sakurai of the first Department of Surgery in 1995, the “Exploratory Committee on Management of Departments” was launched at Jikei University. The method to merge departments was to use the timing at which the chairman for each department reached retirement age and gradually integrated the surgical departments. In April 2001, each independent surgical department was integrated and it marked the end of the multiple surgical systems that had continued for 54 years. In 2002, Professor Teruaki Aoki was appointed as the first Chairman for the large integrated Department of Surgery, following which there was the appointment of Satoshi Kurihara (joint role as Chancellor of Jikei), Professor Katsuhiko Yanaga, and then, from 2007 to date, the current Chairman Professor Takao Ohki. The surgical department for which the founder Kanehiro Takagi was the main driving force has recorded a number of remarkable achievements, but of particular note is that three professors at Jikei University have been appointed as the President of the Japan Surgical Society (JSS) Annual Meeting over the course of more than 119 years. These presidents include Kanehiro Takagi (4th JSS in 1904), Minoru Oi (1965), and Fusahiro Nagao (1986).

## Overview of the current Department of Surgery

Currently, the surgical department is divided into three fields, including respiratory/breast/endocrine surgery (Professor Takashi Otsuka), digestive surgery (Professor Katsuhiko Yanaga), and pediatrics/vascular surgery (Professor Takao Ohki). The Department Chairman is appointed by the Chancellor. Due to the functions of the hospital, the fields are further subdivided and are composed of seven divisions. The chiefs are as follows: pulmonary surgery (Professor Ohtsuka), breast/endocrine surgery (Professor Hiroshi Takeyama), hepatobiliary pancreatic surgery (Professor Katsuhiko Yanaga), upper gastrointestinal tract surgery (Professor Norio Mitsumori), lower gastrointestinal tract surgery (Associate Professor Ken Eto), pediatric surgery (Professor Takao Ohki), and vascular surgery (Professor Takao Ohki) [2].

There are different types of large departments in Japan, but not all are the same. The surgical department at Jikei University is a large department that functions as one department from both the hard and soft aspects. For example, the Department of Internal Medicine at Jikei University is also a large department consisting of nine divisions, the doctor’s private desk is located in nine separate locations, whereas for surgery there is only one office and there is no partitioning between each division within the doctor’s office. Conferences and meetings are held together. Our department operates not only in Jikei University but also is responsible for dispatching surgeons to 38 different affiliated/related hospitals spread out in various areas in Japan. Dispatching surgeons to 38 hospitals is made possible and sustainable by integrating the personnel function as one integrated system supervised by the chairman as opposed to seven different divisions functioning independently as there are always ups and downs in terms of manpower within a small division. Both from the perspective of recruiting personnel and postgraduate education, we are managing integrated surgical resident training programs, and during their 3 years of residency, the residents do not belong to one division but instead immerse themselves in general surgery, only selecting their subspecialty/division after they graduate the surgical residency.

## Unpopularity of surgery in Japan and the turmoil after departmental integration

**Figure 1:**
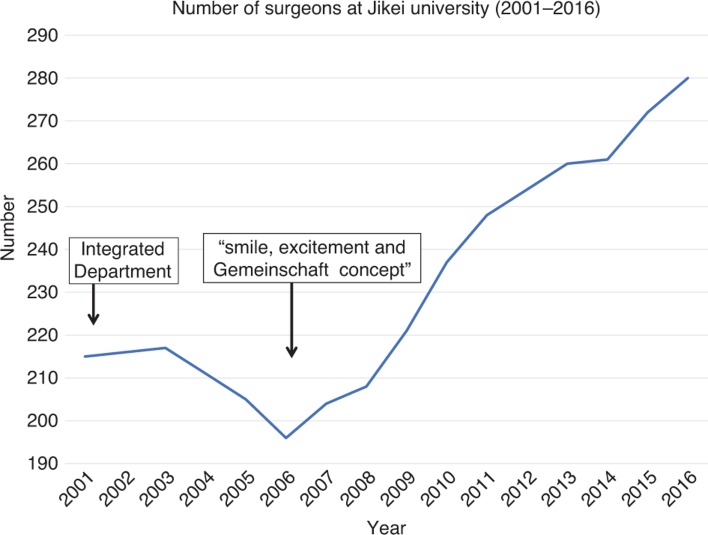
Trend in the number of staff surgeons at Jikei University. One can see the dramatic decrease in the number of surgeons after the surgical department integration and the dramatic recovery after the induction of the excitement and smile concept.

**Figure 2:**
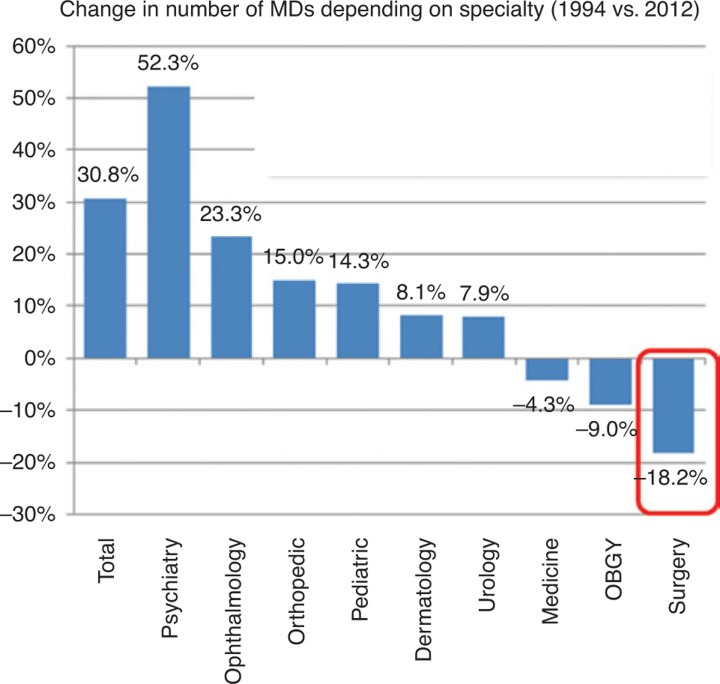
Change in the number/ratio of physicians depending on the specialty in Japan (1994 versus 2012). Over the last 18 years, the number of surgeons has declined by an astonishing 18% due to the younger generation not favoring long work hours and medicolegal risks.

Although there are many advantages of a large department, this did involve, at the same time, a forceful merger of four different cultures and senses of values, and as a result of integrating these previously competing departments under the leadership of the university, it is undeniable that some negative aspects emerged. In fact, in the preceding years before I became the Chairman, due to the aforementioned conflicts and accompanying savage/cold atmosphere and “every man for himself” attitude within the department, more than 10 surgeons were resigning each year, when recruitment of junior residents failed. As a result, the number of staff surgeons declined sharply and was causing trouble not only at Jikei University but also at related hospitals that were dependent on the surgeons who have been dispatched from Jikei University (Figure 1). This shortage of staff occurred not only at Jikei University but also across the nation due to the unpopularity of surgery among medical students and interns (Figure 2) [3]. The unpopularity of surgery was thought to be related to long working hours and medicolegal risks that were unfavored by the younger generation [4], [5], [6]. We fell into a negative spiral where the lack of manpower resulted in excessive working hours, and this in turn caused an even greater decline in recruitment numbers, and as the Department of Surgery had lost its vitality, it was the view of many that integrating previously existing departments into one large department system had ended in failure.

**Figure 3:**
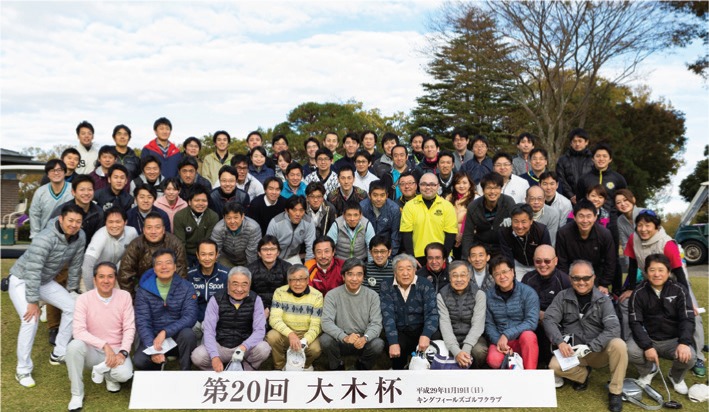
20th Surgical Chairman’s Golf Tournament (The Ohki Cup). In the era of surgical unpopularity, we gathered 84 surgeons at the 20th Ohki Cup Golf Outing. One can imagine the happiness and motivation of the surgeons through their smiles.

**Figure 4:**
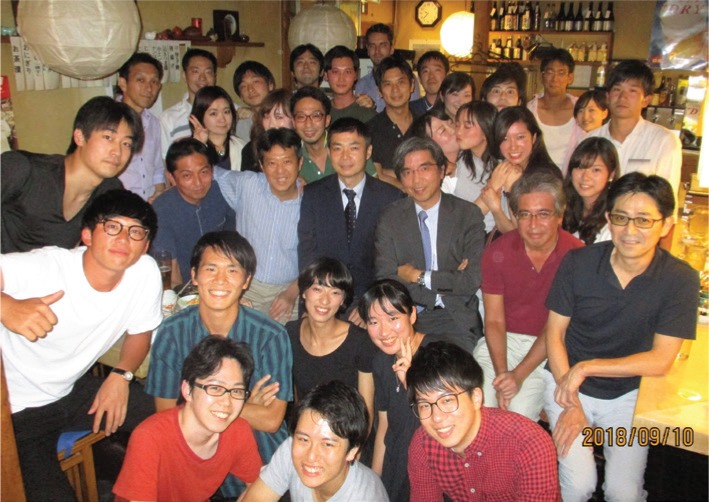
125th Monthly Chairman Invitational Dinner. The Monthly Chairman Invitational Dinner was initiated in 2008 and has been conducted each month consecutively for 12 years in an effort to create an “intimate Gemeinschaft” department. The smiles of the surgeons speak for their happiness and friendship.

In Japan, the salary of hospital employed physicians are more or less the same irrespective of specialty within a given hospital (although they differ between hospitals); therefore, it was not possible to recruit surgeons on an incentive-based program like I did when I was the Professor and Chief of Vascular Surgery at Montefiore Medical Center, Albert Einstein College of Medicine in New York [4], [5], [6]. I was appointed as Chairman of the department in 2007, the year after coming back to Japan, and after having spent 12 years in the United States, I poured my heart and soul into reviving the surgical department [7]. Based on the belief that unless the surgeons’ life is fulfilling and he/she feels happy, we will be unable to provide high-quality medical care as well as recruit young physicians. We took as a cautionary tale the example of the United States where individualism and principles of competition had gone too far and executed a number of reforms, including the revival of the good old Japanese community as the model under the slogan of a “intimate community with excitement and sense of secureness”. The “excitement” aspect can be gained through advanced surgical care that saves lives and delights patients, training of junior staff and students, innovation, research and development, and promotion, etc., all of which are further amplified by the presence of trustworthy colleagues. The “sense of secureness” is created by the department, promising lifelong employment and social stability for individual surgeons irrespective of their performance. In addition, the department and the chairman are responsible for searching reemployment positions for those who have reached retirement age. Together, we referred to it as the “intimate Gemeinschaft”-type organization, which is an antithesis of what I experienced in New York. Furthermore, as surgeons’ happiness could not be realized in a state of manpower shortage, we took plans to recruit medical students by communicating the excitement of surgery to medical students and interns. However, if we eased requirements on surgeons joining the department at mid-career for the purpose of increasing manpower, the order and fairness of the department might erode; therefore, we basically prohibited mid-career entry and established rules that focused on recruiting and training fresh graduates, in other words, Jikei trained and bred. In addition, we abolished various unfair and illogical practices that made working hours unnecessarily longer, introduced a system for the free selection of divisions by residents after completing their surgical residency, allocated budgetary power to the division chiefs, aimed for the transparency and fairness of personnel, and while easing promotion requirements introduced a system in which the senior is evaluated and scored by their subordinates before promotion to make sure that “every man for himself” is not rewarded. We made daily departmental meetings and conferences and journal club fun and interesting with often laughter. Furthermore, we prioritized student education (selected as the most popular department by students 7 years consecutively) and created a surgical department alumni award to recognize contributors. We prohibited “groan and moan” in front of medical students and interns, and we tried to act as their role model through smile and happiness and by saving patients’ lives with innovative procedures. Additionally, as it is difficult to cultivate a sense of belonging and loyalty solely through work relationships alone, we deepened relationship/friendships among staff surgeons by reviving “old fashion” social activities such as departmental trips to the countryside, golf outings (The Ohki Cup; Figure 3), alumni meetings, and the monthly Chairman’s Invitational Dinner, which has thus far been held 130 times (Figure 4). As a whole, we aimed to cultivate a department with “intimate community with excitement and sense of secureness” or in other words “Gemeinschaft with excitement”.

As a result, the number of staff surgeons, which had decreased to 196 members when I was appointed as Chairman, has now increased to 280 surgeons, the highest ever for a surgical department, through double-digit recruitment 10 years straight and a lower number of leavers (Figure 1). Faced with the nationwide unpopularity of surgery in Japan, this achievement has been viewed as miraculous. Owing to these results, the surgical staff is fulfilled at all 38 related hospitals and the number of surgeries as well as revenue has seen a dramatic increase. By creating a system with peace of mind and escaping from the negative spiral, we are entering the positive spiral in which “activity breeds activity” and “people call people”. Thanks to having more personnel available, we constantly have surgeons engaged in basic research and studying abroad and constantly more than 10 surgeons have gone on to graduate school, none of which was possible when we suffered from manpower shortage.

More than anything else, I take pride in increasing our unifying force and a sense of belonging and in other words “Gemeinschaft”-type environment. For example, at the time of the Great East Japan Earthquake, the Ibaragi flood disaster, and the Kumamoto Earthquake, we were not only able to raise donation from the surgical staff but also were able to send several dozen disaster area relief volunteers each time, which is a sign that the department is humanitarian, bonded, and motivated. Additionally, such strength can also be seen from the fact that there was no lack of volunteers for our “rural area surgical support initiative”, which is to dispatch full-time surgeons for 1–2 years to hospitals in Miyagi, Tochigi, Fukushima, and Kochi prefectures, all of which are rural areas.

## Discussion

Shortage of surgeons is not only an issue in Japan but also is a global issue. In the United States, unlike in Japan and Germany, the surgeons are paid well and the shortage is not solely due to unpopularity but is a result of the ceiling of the number of surgical residency slots, which is restricted by the Balanced Budget Act of 1997; therefore, the solution is different [8], [9]. In contrast, Reid-Lombardo et al. conducted a U.S. national survey that revealed that improved reimbursement, lowering practicing cost, tort reform, national loan forgiveness program, and solving lifestyle barriers may be effective in recruiting the younger generation to surgery [10]. With the exception of the lifestyle barrier matter, most issues are U.S. specific and do not directly apply to Japan or Germany and most European Union countries.

If one defines “surgeon shortage” as “general surgeon shortage” due to more young surgeons trained in general surgery advancing into subspecialty fellowships, then the solution may be different [9], [11], [12]. Tierney and Terhune proposed to extend the National Health Service Corps (NHSC) Scholarship Program to include general surgery. Since 1972, this program has been essential in building the primary care workforce in underserved urban and rural locations. The NHSC provides scholarships and stipends to medical students who commit early in their education to residency training in primary care followed by 4 years of service obligation in an approved underserved location [9]. This creative approach may be effective in other countries, including Japan; however, it is a long shot as it requires to create a large enough fund to incentivize surgical trainees to remain as general surgeons. On the contrary, our large department setting is not perfect but is financially less burdensome. Although each staff surgeon at Jikei University has their own subspecialty and focuses on it while working at the university hospital, when they are dispatched to a related hospital that is smaller in size and often in a rural area, more than often they take the subspecialty cap off and practice as a general surgeon. We have mitigated the down side of super specialization with the unified large surgical department system and this approach may be an option where general surgery is in deficit.

A large department system for surgery is rare when looked at from a nationwide perspective, but the attempt at making a surgical department at Jikei University, which could be labeled a social experiment, is receiving attention for the positive results it has produced. The interesting aspect of our revival plan was that it was done in the absence of any financial incentive and was realized purely based on dedication with a smile. Our experience shows that although the younger generation is conscious about quality of life and financial success, they also value excitement, friendship, and happiness, something we were able to provide at the Department of Surgery at Jikei University. However, at the same time, to make it more generalizable, we need to strive to improve the surgeon’s quality of life by creating a better working environment, including sustainable work hours and decent financial incentives.

I would like to take this opportunity to express my appreciation for the University Executive Office, which, having made the bold decision to integrate the surgical departments, saw this through patiently to the end despite the dissenting voices within the department and the headwinds we faced. I would also like to thank our surgical staff who despite feeling the pain of the integration process worked hard for the greater good and the surgical alumni who provided advise and support when we faced interdepartmental conflicts and the potential crisis of the collapse of the departmental integration.

## Conclusions

Our experience showed that although the younger generation is conscious about quality of life and financial success, they also value excitement, friendship, and happiness, something we were able to provide without financial spending. However, our success may be an exception and cannot be generalized; therefore, we should continue to strive to improve the surgeon’s quality of life by creating a better working environment, including sustainable work hours and decent financial incentives.

## Supplementary Material

The article (iss-2019-0005) offers reviewer assessments as supplementary material.

## Article note

Presented at the German Society of Surgery/Japan Surgical Society (GSS/JSS) Joint Session, German Surgical Society, Berlin, Germany, April 18, 2018.
