# Supplementary material for: Reviving surgery with the smile, excitement, and Gemeinschaft concept: attempt at the Department of Surgery, Jikei University
Source: Innov Surg Sci. 2019 Jun 22;4(2):69–74. doi: 10.1515/iss-2019-0005 (PMC6754056; doi:10.1515/iss-2019-0005)
Supplement: Supplementary file 1 [file iss-4-20190005-s001.pdf]

Reviewer Assessment

Takao Ohki*

Reviving surgery with the smile, excitement, and Gemeinschaft concept: attempt at the Department of Surgery, Jikei University

https://doi.org/10.1515/iss-2019-0005
Received April 1, 2019; accepted May 22, 2019

***Corresponding author: Takao Ohki,** Division of Vascular Surgery, Department of Surgery, The Jikei University School of Medicine, Tokyo, Japan, E-mail: takohki@msn.com

Reviewers’ Comments to Original Submission

Reviewer 1: anonymously

Apr 03, 2019

Reviewer Recommendation Term:	Accept
Overall Reviewer Manuscript Rating:	85
Is the subject area appropriate for you?	5 - High/Yes
Does the title clearly reflect the paper’s content?	5 - High/Yes
Does the abstract clearly reflect the paper’s content?	5 - High/Yes
Do the keywords clearly reflect the paper’s content?	5 - High/Yes
Does the introduction present the problem clearly?	5 - High/Yes
Are the results/conclusions justified?	5 - High/Yes
How comprehensive and up-to-date is the subject matter presented?	5 - High/Yes
How adequate is the data presentation?	5 - High/Yes
Are units and terminology used correctly?	5 - High/Yes
Is the number of cases adequate?	N/A
Are the experimental methods/clinical studies adequate?	N/A
Is the length appropriate in relation to the content?	5 - High/Yes
Does the reader get new insights from the article?	5 - High/Yes
Please rate the practical significance.	5 - High/Yes
Please rate the accuracy of methods.	N/A
Please rate the statistical evaluation and quality control.	5 - High/Yes
Please rate the appropriateness of the figures and tables.	5 - High/Yes
Please rate the appropriateness of the references.	5 - High/Yes
Please evaluate the writing style and use of language.	5 - High/Yes
Please judge the overall scientific quality of the manuscript.	5 - High/Yes
Are you willing to review the revision of this manuscript?	Yes

Comments to Author:
Congratulation. In the light of the nationwide problem of unpopularity of surgery related to long and hard working hours and subsequent shortage of creative newcomers in our field the author has addressed an important contribution with a very original and effective solution approach. The results (recruiting young staff and increasing the number of surgeons in his large department system of surgery in absence of financial incentive) are convincing. His concept of “reviving surgery with the smile, excitement and Gemeinschaft” set the right focuses. However it is not enough to celebrate such rare positive concepts: we must strive to emulate them.

Open Access. © 2019 Ohki T., published by De Gruyter. This work is licensed under the Creative Commons Attribution 4.0 Public License.

Reviewer 2: Matthias Anthuber, M.D.

Apr 14, 2019

**Reviewer Recommendation Term:**	Accept with Minor Revision
**Overall Reviewer Manuscript Rating:**	85
Is the subject area appropriate for you?	5 - High/Yes
Does the title clearly reflect the paper's content?	5 - High/Yes
Does the abstract clearly reflect the paper's content?	5 - High/Yes
Do the keywords clearly reflect the paper's content?	3
Does the introduction present the problem clearly?	4
Are the results/conclusions justified?	4
How comprehensive and up-to-date is the subject matter presented?	5 - High/Yes
How adequate is the data presentation?	3
Are units and terminology used correctly?	5 - High/Yes
Is the number of cases adequate?	N/A
Are the experimental methods/clinical studies adequate?	3
Is the length appropriate in relation to the content?	5 - High/Yes
Does the reader get new insights from the article?	5 - High/Yes
Please rate the practical significance.	4
Please rate the accuracy of methods.	4
Please rate the statistical evaluation and quality control.	4
Please rate the appropriateness of the figures and tables.	4
Please rate the appropriateness of the references.	4
Please evaluate the writing style and use of language.	3
Please judge the overall scientific quality of the manuscript.	4
Are you willing to review the revision of this manuscript?	Yes

Comments to Author:
The paper briefly outlines the development of the surgical department at the Jikei University in Japan and describes the approach of the University to tackle the shortage in surgical staff and the lack of recruit surgeons. The author explains in detail how they achieved a significant increase in staff and improved the satisfaction and happiness of employees without financial incentives. He gives an inside into the three main components of their concept – smile, excitement and a feeling of belonging and empathy ("Gemeinschaft").
The presented developmental figures regarding the increase in staff are impressive and the story behind this success is written in a personal and empathic way. Given the shortage of young surgeons and the missing interest in surgery by medical students in almost all countries the topic is internationally of high socio-political relevance.
Given the fact, that employee satisfaction was an imminent component of the presented concept itself ("smile") and of the desired outcome, evaluation data regarding this topic is missing. Although the impressive increase in staff over the years seems to be a proof of concept.
Grammar and punctuation need some improvements from a native English speaking person.
In summary the topic of shortage of surgeons has been internationally discussed in the last years, proven by several publications. It would increase the quality of the paper if the own experiences would be discussed in the light of the existing literature and current knowledge.

Major Issues
- Grammar and punctuation need improving

Minor Issues
- From a scientific point of view the topic seems of minor interest
- Evaluation data regarding the employee satisfaction is missing.
- How do the findings of the paper fit into the context of the existing literature and current knowledge?

Appendix
Predicted shortage of vascular surgeons in the United Kingdom: A matter for debate?
Harkin DW, Beard JD, Shearman CP, Wyatt MG.
Surgeon. 2016 Oct;14(5):245-51. doi: 10.1016/j.surge.2015.10.004. Epub 2015 Dec 2.
PMID: 26654693

Expanding the National Health Service Corps Scholarship Program to General Surgery: A Proposal to Address the National Shortage of General Surgeons in the United States.
Tierney J, Terhune K.
JAMA Surg. 2017 Apr 1;152(4):315-316. doi: 10.1001/jamasurg.2016.4996. Review. No abstract available.
PMID: 28097331

Ten-year reassessment of the shortage of general surgeons: Increases in graduation numbers of general surgery residents are insufficient to meet the future demand for general surgeons.
Ellison EC, Pawlik TM, Way DP, Satiani B, Williams TE.
Surgery. 2018 Oct;164(4):726-732. doi: 10.1016/j.surg.2018.04.042. Epub 2018 Aug 8.
PMID: 30098811

Gazing into the crystal ball: Preventing the inevitable shortage of cardiothoracic surgeons.
Nguyen TC.
J Thorac Cardiovasc Surg. 2018 Feb;155(2):830-831. doi: 10.1016/j.jtcvs.2017.09.105. Epub 2017 Oct 3. No abstract available.
PMID: 29061466

Workforce shortage for general surgeons: results from the Society for Surgery of the Alimentary Track (SSAT) surgeon shortage survey.
Reid-Lombardo KM, Glass CC, Marcus SG, Liesinger J, Jones DB; Public Policy and Advocacy Committee of the SSAT.
J Gastrointest Surg. 2014 Dec;18(12):2061-73. doi: 10.1007/s11605-014-2636-8. Epub 2014 Sep 23.
PMID: 25245765

Maximizing Capacity in Times of Shortage: A Proposal for Surgeon Volunteerism in the US.
Ghomrawi HM.
Ann Surg. 2016 Jun;263(6):1073-4. doi: 10.1097/SLA.0000000000001521. No abstract available.
PMID: 27172101

Authors’ Response to Reviewer Comments

Apr 28, 2019

Reviewers’ comments:

Reviewer #1:

Congratulation. In the light of the nationwide problem of unpopularity of surgery related to long and hard working hours and subsequent shortage of creative newcomers in our field the author has addressed an important contribution with a very original and effective solution approach. The results (recruiting young staff and increasing the number of surgeons in his large department system of surgery in absence of financial incentive) are convincing. His concept of “reviving surgery with the smile, excitement and Gemeinschaft” set the right focuses. However it is not enough to celebrate such rare positive concepts: we must strive to emulate them.

1) Response. I appreciate your kind comments. Thank you.
2) “However it is not enough to celebrate such rare positive concepts: we must strive to emulate them.” i totally agree with this comment and thus have made changes in the manuscript. in response to this comment, I have added the following line in the abstract and the conclusion.

Page 1, abstract and page 6 conclusion. Added line.

“However, our success may be an exception and cannot be generalized and therefore we should continue to strive for improved surgeon’s quality of life by creating a better working environment including sustainable work hours and decent financial incentives.”

Reviewer #2:

The paper briefly outlines the development of the surgical department at the Jikei University in Japan and describes the approach of the University to tackle the shortage in surgical staff and the lack of recruit surgeons. The author explains in detail how they achieved a significant increase in staff and improved the satisfaction and happiness of employees without financial incentives. He gives an inside into the three main components of their concept – smile, excitement and a feeling of belonging and empathy (“Gemeinschaft”).

The presented developmental figures regarding the increase in staff are impressive and the story behind this success is written in a personal and empathic way. Given the shortage of young surgeons and the missing interest in surgery by medical students in almost all countries the topic is internationally of high socio-political relevance.
Given the fact, that employee satisfaction was an imminent component of the presented concept itself ("smile") and of the desired outcome, evaluation data regarding this topic is missing. Although the impressive increase in staff over the years seems to be a proof of concept.
Grammar and punctuation need some improvements from a native English speaking person.
In summary the topic of shortage of surgeons has been internationally discussed in the last years, proven by several publications . It would increase the quality of the paper if the own experiences would be discussed in the light of the existing literature and current knowledge.

Major Issues
- Grammar and punctuation need improving

Minor Issues
- From a scientific point of view the topic seems of minor interest
- Evaluation data regarding the employee satisfaction is missing.
- How do the findings of the paper fit into the context of the existing literature and current knowledge?

Response
1) Comment #1. "Grammar and punctuation need improving" I have tried my best to improve the English grammar and punctuation throughout the manuscript. Please see redline function of the revised manuscript. I would like to rely on the professional editing of the publisher.
2) Comment #2. "From a scientific point of view the topic seems of minor interest" although I agree with this comment, I was given this theme and title and was invited to submit this article and thus, cannot do anything about it. If the editors feels so, please reject this article.
3) Comment #3." Evaluation data regarding the employee satisfaction is missing" this is also a valid critique. Although I am not able to objectively prove that employee satisfaction has improved, in the view of the fact that many young surgeons joined our department in the absence of financial incentives or increase in salary, I believe that the fact that we were successful in recruiting young surgeons as shown in figure 1 is a circumstantial evidence that the staff surgeons are satisfied and happy. Otherwise, there are no other explanation for the success in young surgeon recruitment.
4) Comment #4. I appreciate for informing me of these related articles. I have quoted them and added the following paragraph in the discussion section, page 5-6.

"Shortage of surgeon is not only an issue in Japan but also is a global issue. In the US, unlike in Japan and Germany, the surgeons are paid well and the shortage is not solely due to unpopularity but is a result of ceiling of the number of surgical residency slots which is restricted by the Balanced Budget Act of 1997 and therefore the solution is different.8,9 On the other hand, Reid-Lombardo KM et al conducted a US national survey which revealed that improved reimbursement, lowering practicing cost, tort reform, national loan forgiveness program and solving lifestyle barriers may be effective in recruiting younger generation to surgery.10 With the exception of lifestyle barrier matter, most issues are US specific and do not directly apply to Japan or Germany and most EU countries.
If one defines "surgeon shortage" as "general surgeon shortage" due to more young surgeons trained in general surgery advancing into subspecialty fellowships, then the solution may be different.9,11,12 Tierney J. et al proposes to extend the National Health Service Corps (NHSC) scholarship program to include general surgery. Since 1972, this program has been essential in building the primary care workforce in underserved urban and rural locations. NHSC provides scholarships and stipends to medical students who commit early in their education to residency training in primary care, followed by 4 years of service obligation in an approved underserved location.9 This creative approach may be effective in other countries including Japan, however, it is a long shot since it requires to create a large enough fund to incentivize surgical trainees to remain as general surgeons. On the contrary, our large department setting is not perfect but financially less burdensome. Although each staff surgeons at Jikei university has their own subspecialty and focuses on it while working at the university hospital, when they are dispatched to a related hospital which is smaller in size and often in rural area, more than often, they take the subspecialty cap off and practice as a general surgeon. We have mitigated the down side of super specialization with the unified large surgical department system and this approach may be an option where general surgery is in deficit."
